# 300. Disturbance of Gut Microbial Diversity in ICU Patients from Colombia is Driven by Multi-Drug Resistant Organisms (MDRO) Colonization

**DOI:** 10.1093/ofid/ofad500.372

**Published:** 2023-11-27

**Authors:** Alexandra Parada Torres, Betsy Castro, Thomas Malavet, Nicolas Forero, Juan Matiz, Yordy Rodriguez, Luis Zambrano, Karen Melissa Ordoñez, Alejandra Santamaria, Fidel Ospina, Jaime Beltran, Fredy Guevara, Juliana Martinez, Alexandra Porras, Paola Sarmiento, Luisa Aroca, Alejandra Mora, Cesar A Arias, Lina Paola Carvajal, Sandra Rincon, Jinnethe Reyes

**Affiliations:** Universidad el Bosque, Bogotá, Distrito Capital de Bogota, Colombia; Universidad el Bosque, Bogotá, Distrito Capital de Bogota, Colombia; Universidad el Bosque, Bogotá, Distrito Capital de Bogota, Colombia; Universidad el Bosque, Bogotá, Distrito Capital de Bogota, Colombia; Universidad el Bosque, Bogotá, Distrito Capital de Bogota, Colombia; Universidad el Bosque, Bogotá, Distrito Capital de Bogota, Colombia; Universidad el Bosque, Bogotá, Distrito Capital de Bogota, Colombia; ESE Hospital Universitario San Jorge de Pereira, Pereira, Risaralda, Colombia; Hospital San Jorge de Pereira, Bogotá, Distrito Capital de Bogota, Colombia; Clinica Colsubsidio Calle 100, Bogotá, Distrito Capital de Bogota, Colombia; Clinica Colsubsidio Calle 100, Bogotá, Distrito Capital de Bogota, Colombia; Fundacion Santa Fe de Bogota, Bogotá, Distrito Capital de Bogota, Colombia; Universidad el Bosque, Bogotá, Distrito Capital de Bogota, Colombia; Los Cobos Medical Center, Bogotá D.C., Distrito Capital de Bogota, Colombia; Los Cobos Medical Center, Bogotá D.C., Distrito Capital de Bogota, Colombia; Clinica General del Norte, Barranquilla, Atlantico, Colombia; Universidad el Bosque, Bogotá, Distrito Capital de Bogota, Colombia; Houston Methodist and Weill Cornell Medical College, Houston, TX; Universidad El Bosque, Bogotá D.C., Distrito Capital de Bogota, Colombia; Universidad El Bosque, Bogotá D.C., Distrito Capital de Bogota, Colombia; Universidad El Bosque, Bogotá D.C., Distrito Capital de Bogota, Colombia

## Abstract

**Background:**

Gut microbiota plays a critical role in health, with essential functions in physiology, metabolism, immunity, regulation, and protection against pathogens. Critically-ill patients admitted on the ICU experience perturbation in the microbiota, with reduction in the phylogenetic diversity and composition. Here, we aimed to investigate the rectal microbiota in patients admitted on the ICU from Colombia, focused on MDRO colonization.

**Methods:**

Microbiome analysis and microbiological screening for MDRO and WGS were performed in rectal swabs taken on admission day from 180 patients from 5 ICUs. Microbiome analysis was determined by 16S rRNA sequencing using Illumina Miseq platform. alpha-diversity metrics were calculated by Richness, Simpson, and Shannon indexes, and beta-diversity was determined using Similarity Percentages (SIMPER) analysis. Patients were grouped into 2 cohorts: MDRO-colonized and non-colonized. Clinical data was collected using REDCap.

**Results:**

MDRO colonization was detected in 13 patients (7%), and *Enterobacter cloacae* complex was the most prevalent microorganism (38%), followed by *K. pneumoniae* (23%), and *P. aeruginosa* (15%). Of these MDRO, 23% and 15% harbored KPC-2 and KPC-3/OXA-9 combination, respectively. *Prevotella* and *Bacteroides* were the most abundant genus in the microbiota of all patients. Interestingly, microbiota of MDRO-colonized patients showed lower diversity (Shannon *p*:0.0025, Simpson *p:* 0.0031 and richness 0.012) and had less bacterial taxa than the observed in the non-colonized group (n=167), in which *Bacteroides, Porphyromonas, Campylobacter,* and *Oscillospiraceae genus* were exclusively detected. Moreover, *Parabacteroides* was a genus exclusively identified in the MDRO colonized group. Further, 14 genus including *Oscillospiraceae, Lactobacillus,* and *Faecalibacterium* related to beneficial functions were lower in abundance from MDRO-colonized patients, whereas some pathobionts were present in high abundance in this group. Most of patients were men (58%); median age was 59 y/o. Charlson comorbidity index 4 (IQR=1-6).

**Conclusion:**

Loss in microbial diversity, with low abundance of beneficial microorganisms were associated with MDRO gut colonization in ICU patients from Colombia.

**Disclosures:**

**Karen Melissa Ordoñez, n/a**, Pfizer: Advisor/Consultant|Pfizer: Honoraria

